# Chronic heart failure is characterized by altered mitochondrial function and structure in circulating leucocytes

**DOI:** 10.18632/oncotarget.26164

**Published:** 2018-10-12

**Authors:** Roberta Coluccia, Salvatore Raffa, Danilo Ranieri, Andrea Micaloni, Sabatino Valente, Gerardo Salerno, Cristina Scrofani, Marco Testa, Giovanna Gallo, Erika Pagannone, Maria Rosaria Torrisi, Massimo Volpe, Speranza Rubattu

**Affiliations:** ^1^ IRCCS Neuromed, Pozzilli, Isernia, Italy; ^2^ Department of Clinical and Molecular Medicine, School of Medicine and Psychology, Sapienza University of Rome, Sant’Andrea University Hospital, Rome, Italy; ^3^ Ultrastructural Pathology Lab-Medical Genetics and Advanced Cellular Diagnostics Unit, Sant’Andrea University Hospital, Rome, Italy; ^4^ Cardiology Unit, Sant’Andrea University Hospital, Rome, Italy

**Keywords:** heart failure, peripheral blood mononuclear cells, mitochondrial dysfunction, oxidative stress, mitophagy, Pathology

## Abstract

Oxidative stress is currently viewed as a key factor in the genesis and progression of Heart Failure (HF). The aim of this study was to characterize the mitochondrial changes linked to oxidative stress generation in circulating peripheral blood mononuclear cells isolated from chronic HF patients (HF_PBMCs) in order to highlight the involvement of mitochondrial dysfunction in the pathophysiology of HF.

To assess the production of reactive oxygen species (ROS), mitochondrial function and ultrastructure and the mitophagic flux in circulating PBMCs we enrolled 15 patients with HF and a control group of ten healthy subjects. The HF_PBMCs showed a mitochondrial population consisting of damaged and less functional organelles responsible of higher superoxide anion production both at baseline and under *in vitro* stress conditions, with evidence of cellular apoptosis. Although the mitophagic flux at baseline was enhanced in HF_PBMCs at level similar to those that could be achieved in control PBMCs only under inflammatory stress conditions, the activation of mitophagy was unable to preserve a proper mitochondrial dynamics upon stress stimuli in HF.

In summary, circulating HF_PBMCs show structural and functional derangements of mitochondria with overproduction of reactive oxidant species. This mitochondrial failure sustains a leucocyte dysfunctional status in the blood that may contribute to development and persistence of stress conditions within the cardiovascular system in HF.

## INTRODUCTION

Heart failure (HF) is a clinical syndrome affecting more than 23 millions of patients worldwide with a high associated morbidity and mortality [1]. Over the last 30 years several improvements in treatments favored survival in these patients but the prognosis remains unsatisfactory.

In order to better understand HF and to identify the best therapeutic options, it is important to fully investigate the underlying pathophysiological mechanisms.

Oxidative stress and chronic inflammation are key factors involved in the pathophysiology of HF [2]. In this context, the role of mitochondrial dysfunction appears to be of increasing importance [3].

Mitochondria are the main source of intracellular ATP. They also control cell death and survival by integrating information from multiple signaling pathways [4]. The mitochondrial network exhibits high tissue specificity in relation with the cell functions. Any change of the internal organization of mitochondria could impair cell energetics and, as a consequence, cell function and viability [5].

The cardiac mitochondria produce 90% of the required ATP. They occupy one third of the cell volume, are embedded in a dense and complex organization and are able to adjust their morphology and location depending on the energy need and the metabolic status [6].

To ensure the maintenance of the morpho-functional integrity of the mitochondrial network and to mantain the normal cell function and tissue homeostasis, a quality control of the mitochondrial structure is essential [7]. In fact, mitochondria are constantly under dynamic events to adapt themselves toward changes in the cellular environment. Specifically, the full equilibrium between mitochondrial fusion and fission, together with the elimination of dysfunctional mitochondria and mitochondrial biogenesis, represents a crucial mechanism to ensure mitochondrial homeostasis [8, 9].

Fusion of reversibly damaged mitochondria with healthy ones can favor their functional repair. Fission allows the maintenance of functional mitochondria by segregating damaged parts. However, when mitochondria incur irreversible damage, fission leads to selective elimination of the damaged organelles by mitochondrial-specific autophagy, referred to as mitophagy [10].

Oxidative damage contributes to impairment of the electron transport chain, to mitochondrial uncoupling, and, ultimately, to bioenergetic dysfunction characterized by reduced ATP production and further accumulation of ROS [11]. Finally, oxidative stress is associated with mitochondrial transition pore opening, which promotes cell death [12]. At ultrastructural level, the findings of mitochondrial suffering were related to a progressive reduction of mitochondrial surface associated with intact cristae and relevant reduction of internal mitochondrial membrane (IMM) length with respect to outer mitochondrial membrane (OMM), namely IMM/OMM index [13, 14].

In the presence of cellular damage caused by excessive ROS production from aberrant mitochondria, the cells activate mitophagy [12, 15, 16]. This process involves the activation of PTEN induced kinase 1 (PINK1) and of parkin [12].

Mitochondrial dysfunction has been previously reported in dilated cardiomyopathy [17] and in failing hearts [18]. Experimental studies in animals with pressure overload-induced left- and right- ventricular failure show increased mitochondrial ROS generation [19, 20]. The myocardial oxidative stress deteriorates cardiac function by inducing cellular damage and death [21]. Interestingly, the role of mitochondrial dysfunction present in circulating peripheral blood mononuclear cells (PBMCs) has been previously involved in the pathophysiology of HF [22–24]. Moreover, the role of reactive oxygen species generated by PBMCs has been explored in patients with cardiovascular disease [25] and HF [24]. Surprisingly, the available data show that PBMCs undergo changes similar to failing cardiomyocytes in HF [26]. In fact, studies on leukocyte mitochondria in advanced and decompensated congestive HF [22] or after cardiac surgery [27] show that the degree of mitochondrial dysfunction is associated with the disease severity and that this alteration gradually recovers after clinical stabilization. However, a fine characterization of mitochondrial changes linked to oxidative stress generation in circulating PBMCs isolated from chronic HF is still lacking.

The purpose of this study was to characterize the involvement of mitochondrial dysfunction in the pathophysiology of HF by analyzing the mitochondrial structural and functional changes in relation to cell damage in circulating PBMCs isolated from chronic HF patients (HF_PBMCs).

## RESULTS

### Characteristics of the study population

The main clinical and laboratory characteristics of the subjects included into the study are summarized in Table 1. In the HF group, all patients had chronic HF; 14 of them presented with NYHA class I-II, 1 patient with stable NYHA class III. Among the HF patients, 14 had idiopathic dilated cardiomyopathy and one patient had history of myocarditis. Furthermore, five HF patients had associated renal failure.

**Table 1:** Clinical and laboratory characteristics of HF patients and control subjects

	HF group (Mean ± SD)	CTRL group (Mean ± SD)	Statistics
**Age (years)**	56.6 ± 10.8	49.3 ± 8	*p* = 0.10
**Male sex (%)**	80	80	*p* = 1
**BMI (kg/m^2^)**	28.1 ± 3.2	24.1 ± 2.4	*p* < 0.05
**NYHA class**	I–II: 14 patients III: 1 patient	0	-
**SBP/DBP (mmHg)**	108 ± 2.5/65 ± 3	116 ± 2.5/70 ± 3	*p* = 0.26
**LVEF (%)**	28 ± 8	65 ± 2	*p* < 0.00001
**LVEDD (mm)**	67 ± 10	48 ± 3	*p* < 0.0001
**LA size (mm)**	48 ± 10	36 ± 2	*p* < 0.01
**Creatinine (mg/dL)**	1.19 ± 0.38	0.84 ± 0.2	*p* < 0.05
**BUN (mg/dl)**	27.8 ± 16.7	14.9 ± 1.9	*p* < 0.05
**Hemoglobin (g/dl)**	14.1 ± 1.7	14.8 ± 0.5	*p* = 0.2
**BNP (pg/ml)**	451 ± 672	NA	-
**Number of subjects**	15	10	-

Abbreviations: LVEF: Left Ventricular Ejection Fraction, LVEDD: Left Ventricular End Diastolic Diameter, LA: Left Atrium, SBP/DBP: Systolic Blood Pressure/Diastolic Blood Pressure, NA: Not Assessed.

### The PBMCs from HF patients are characterized by mitochondrial impairment and higher levels of oxidative stress

**Figure 1:**
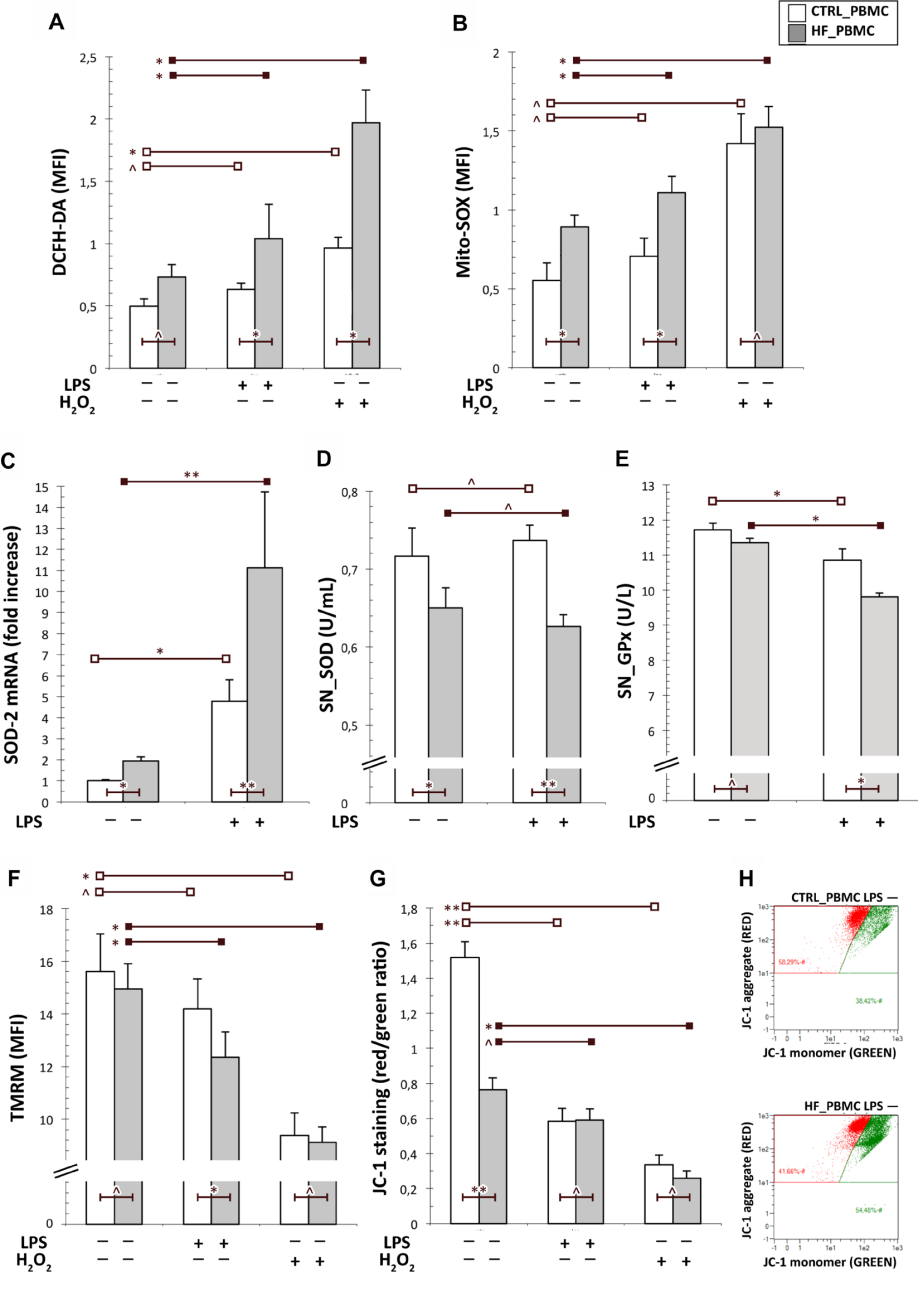
Analysis of the mitochondrial function in PBMCs from HF and CTRL subjects (**A**, **B**) Cytofluorimetric assay of cytoplasmic and mitochondrial ROS generation in unstimulated or stimulated PBMCs with LPS. The mitochondrial ROS levels were higher in PBMCs from HF respect to CTRL both at baseline and after LPS stimulation. As expected, the stimulation with H2O2 increased both cytoplasmic and mithocondrial oxidative stress particularly in HF_PBMCs. (**C**) A significant upregulation of the SOD-2 mRNA levels, as detected by RT-PCR, was found in HF_PBMCs with respect to CTRL_PBMCs, particularly after LPS stimulation (Mann–Whitney test: ^*^p < 0.05, ^**^p < 0.01 and ^^^*p* = NS). (**D**, **E**) Assessment of antioxidant enzymes activity in supernatants of PBMCs cultures; the SOD and GPx activities were decreased in the HF_PBMCs compared to CTRL_PBMC (Student *T* test: ^*^p < 0.05, ^**^p < 0.01 and ^^^*p* = NS). (**F**–**H**) Cytofluorimetric assay for mitochondrial membrane potential (Δψm; TMRM staining) and mitochondrial depolarization index (JC-1 staining) in unstimulated or stimulated PBMCs with LPS. The data reflect a significant mitochondrial depolarization in HF_ with respect to CTRL_PBMCs. (Student *T* test: ^*^p < 0.05, ^**^p < 0.01 and ^^^*p* = NS). The panel G shows a JC-1 staining plot from representative PBMCs samples.

**Figure 2:**
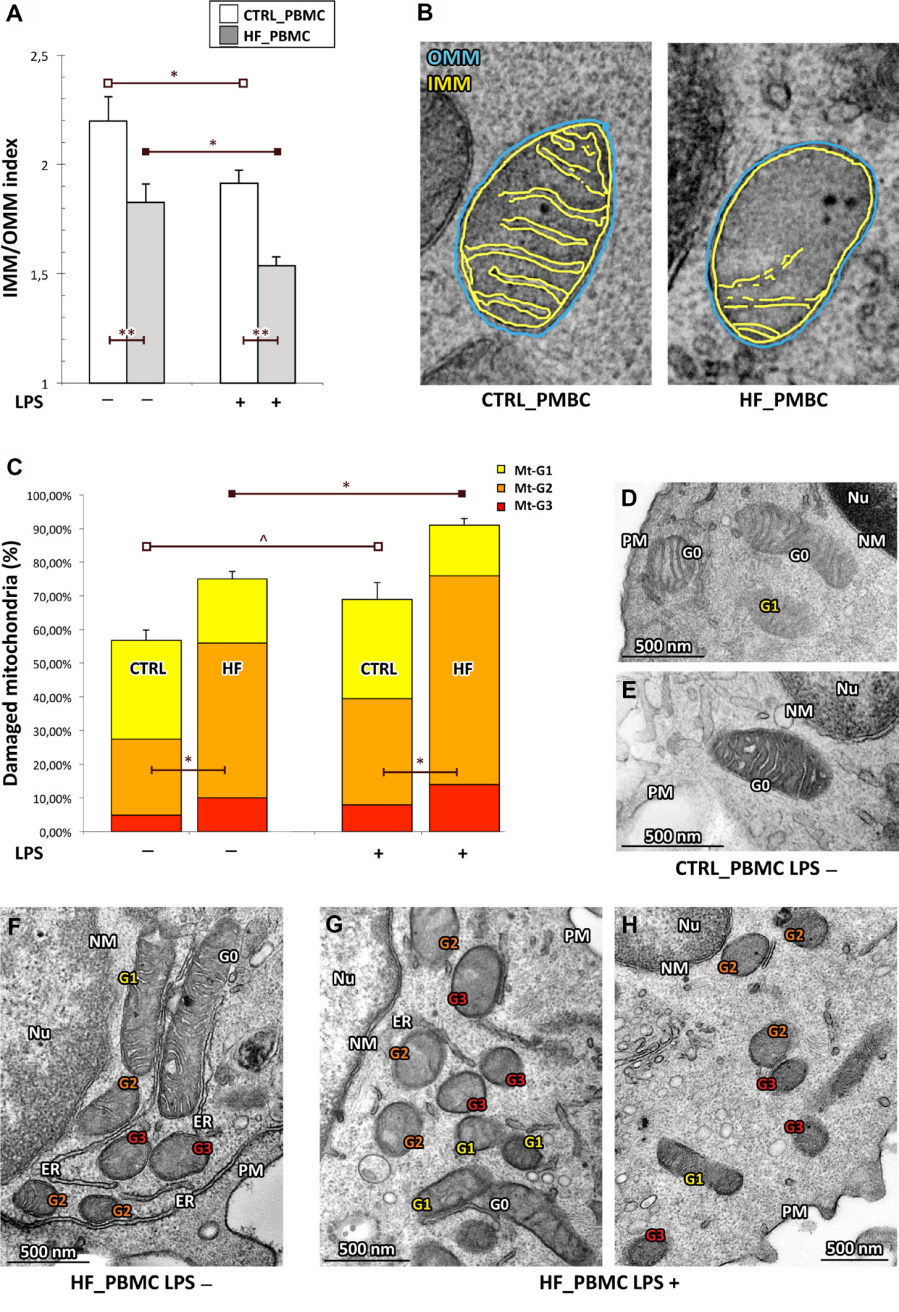
Quantitative analysis of ultrastructural damage in mitochondria from HF_ and CTRL_PBMCs (**A**, **B**) Graphical representation of the IMM/OMM values. By applying the IMM/OMM index associated with convolution loss of inner mitochondrial membrane, we observed a significantly lower index values in the HF_ compared to CTRL_PBMCs at baseline condition with a further worsening after LPS-stimulation (Student *T* test: ^*^p < 0.05 and ^**^p < 0.01). Representative micrographs are displayed in B. (**C**) Graphical representation of the ultrastructural Mt-grading of damage. At baseline, the burden of overall damage was higher in HF_ with respect to CTRL_PBMCs; the standardized pro-inflammatory stimulus produced a strong increase of the mitochondrial overall damage both in the HF_ and CTRL_PBMCs (Student *T* test; ^*^p < 0.05, ^**^p < 0.01 and ^^^*p* = NS). (**D**–**H**) Representative micrographs of ultrastructural damage in mitochondria from human HF_ and CTRL_PBMCs. The burden of mitochondrial damage after LPS stimulation was higher particularly in HF_PBMCs (G–H); the mitochondria were characterized by degeneration of convolutions related to lack of the inner membrane and subsequently by reduction of mitochondrial area with intact cristae. The mitochondria of PBMCs obtained from healthy subjects showed mitochondria with normal morphology or only with slight damage after stimulation (D and E). The mitochondria of unstimulated HF_PBMCs displayed a pattern of intermediate damage (F). (TEM micrographs, uranyl acetate/lead citrate; Legend: NM, nuclear membrane, PM, plasma membrane; Nu, nucleus; ER, endoplasmic reticulum Gx: grade of mitochondrial damage).

In order to analyze the mithocondrial function of HF_PBMCs with respect to PBMCs, we assessed levels of Δѱm and of cytoplasmic and mithocondrial ROS. The baseline level of cytoplasmic ROS did not show significant differences in the HF_PBMCs compared with the CTRL_PBMCs, though the proinflammatory stimulus with LPS induced a significant increase of cytoplasmic ROS in the HF_PBMCs (Figure 1A; *p* < 0.05). In contrast, the mitochondrial ROS levels detected with the MitoSOX Red were higher in the HF_PBMCs with respect to the CTRL_PBMCs, both at baseline condition and after LPS stimulation (Figure 1B; *p* < 0.05). As expected, the stimulation with H_2_O_2_ increased both cytoplasmic and mithocondrial oxidative stress particularly in HF_PBMCs (Figure 2A–2B; *P* < 0.05).

The higher oxidative stress production in HF_PBMCs was also supported by the activation of counteracting pathways culminating with the upregulation of antioxidant genes. In fact, we found a significant difference of mRNA synthesis of Mn-superoxide dismutase (SOD-2) with respect to CTRL_PBMCs (Figure 1C; *p* < 0.05). In contrast, SOD activity was significantly decreased in the HF_PBMCs (Figure 1D; *p <* 0.05 and < 0.01). The GPx activity was also reduced in HF_PBMCs treated with the proinflammatory stimulus (Figure 1E; *p <* 0.05). This result would suggest either accelerated degradation or inactivation of the antioxidant mitochondrial enzymes during a stress condition [28].

The cytofluorimetric analysis of mitochondrial membrane potential by TMRM and JC-1 staining highlighted significant differences between the PBMCs groups, particularly following LPS stimulation, reflecting a significant mitochondrial depolarization in HF_PBMCs (Figure 1F–1H; *p <* 0.05).

Based on the above described functional data, we quantified the ultrastructural changes in mitochondria from HF and CTRL_PBMCs samples, stimulated or not as above, by two parameters of morphological damage: i) the percentage of mitochondrial area carrying intact cristae; and ii) the loss of IMM assessed as the IMM/OMM index. By applying the IMM/OMM index associated with convolution loss of inner mitochondrial membrane, we observed a significantly lower index values in the HF_PBMCs compared to CTRL_PBMCs at baseline with a further worsening after LPS-stimulation (Figure 2A–2B; *p <* 0.01).

When using the grading scale of mitochondrial damage (Mt-G), HF_PBMCs showed a burden of overall damage, based on the distribution of Mt-G1 to Mt-G3 levels, significantly higher with respect to CTRL_PBMCs both at baseline and after LPS stimulation (Figure 2C; *p <* 0.05). At the ultrastructural level the CTRL_PBMCs showed mitochondria with normal morphology or only with slight damage after stimulation (Figure 2D–2E). In contrast, the HF_PBMCs presented degeneration of convolutions related to lack of the IMM and also reduction of mitochondrial area with intact cristae (Figure 2F–2H).

**Figure 3:**
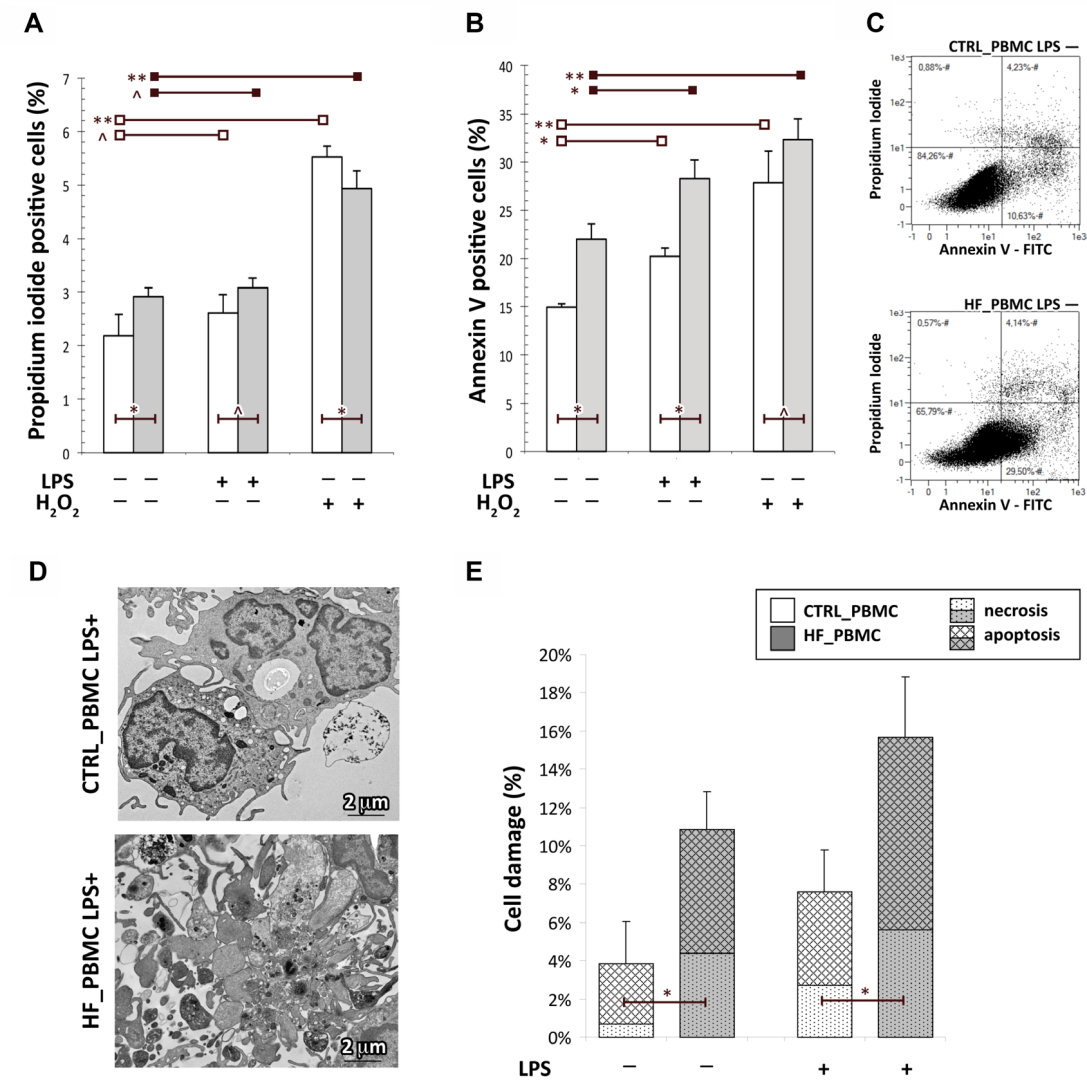
Assessment of cell death in PBMCs obtained from HF and CTRL subjects (**A**–**C**) The FACS analysis with Annexin V-FITC/PI staining showed that the percentage of apoptotic cells in HF_PBMCs was significantly higher with respect to CTRL_PBMCs, particularly after LPS-stimulation (Student *T* test: ^*^p < 0.05, ^**^p < 0.01 and ^^^*p* = NS). The panel C shows a flow cytometric plot for Annexin/PI staining from representative PBMCs samples. (**D** and **E**) Ultrastructural quantitative evaluation of cell damage. The HF_PBMCs displayed a higher percentage of features of cell damage with respect to CTRL_PBMCs (Mann–Whitney test: ^*^p < 0.05). Representative micrographs of LPS-stimulated PBMCs (panel D) show the cytoplasmic vacuolization and apoptotic fragmentation characteristic of the necrotic or apoptotic cell death, respectively. (TEM micrographs, uranyl acetate/lead citrate.

It is known that the preservation of mitochondrial integrity and function is critical for cell physiology. In response to various environmental stresses, cellular energy production can be disrupted and mitochondria become major producers of partially reduced species of molecular oxygen, which cause cellular damage also triggering the apoptotic pathway [7, 29–31]. To evaluate if the mitochondrial impairment in HF_PBMCs can also induce cytotoxic effects, cell damage was evaluated using annexin-V/PI staining. The flow cytometric analysis showed that the percentage of apoptotic cells in HF_PBMCs was significantly higher with respect to CTRL_PBMCs (Figure 3A–3C; *p <* 0.05). To confirm these data, an ultrastructural quantitative evaluation of cell damage was performed. Transmission electron microscopy revealed that the HF_PBMCs showed several features of cellular damage, such as apoptotic nuclei with areas of marginal, dense-stained chromatin and, less frequently, caryorrexis, caryolisis and fragmentation of cellular membranes (Figure 3D–3E; *p <* 0.05).

Altogether, our data indicate that the HF_PBMCs are characterized by mitochondrial dysfunction with increased generation of mitochondrial ROS and that, as expected, these conditions are associated with a strong alteration of mitochondrial ultrastructure and with features of cellular damage. In addition, the standardized proinflammatory stimulation with LPS unmasked the mitochondrial failure that may not be overt at baseline condition.

### The PBMCs from HF patients show an extensive activation of the mitophagic process similar to that of the PBMCs from healthy subjects upon inflammatory stress

As described above, in the presence of excessive ROS generation from aberrant mitochondria, the cells activate mitophagy. When mitophagy is triggered, a significant reduction of the mitochondrial pool is observed and the functionally compromised mitochondria appear smaller, rounded and with loss of internal cristae [32].

**Figure 4:**
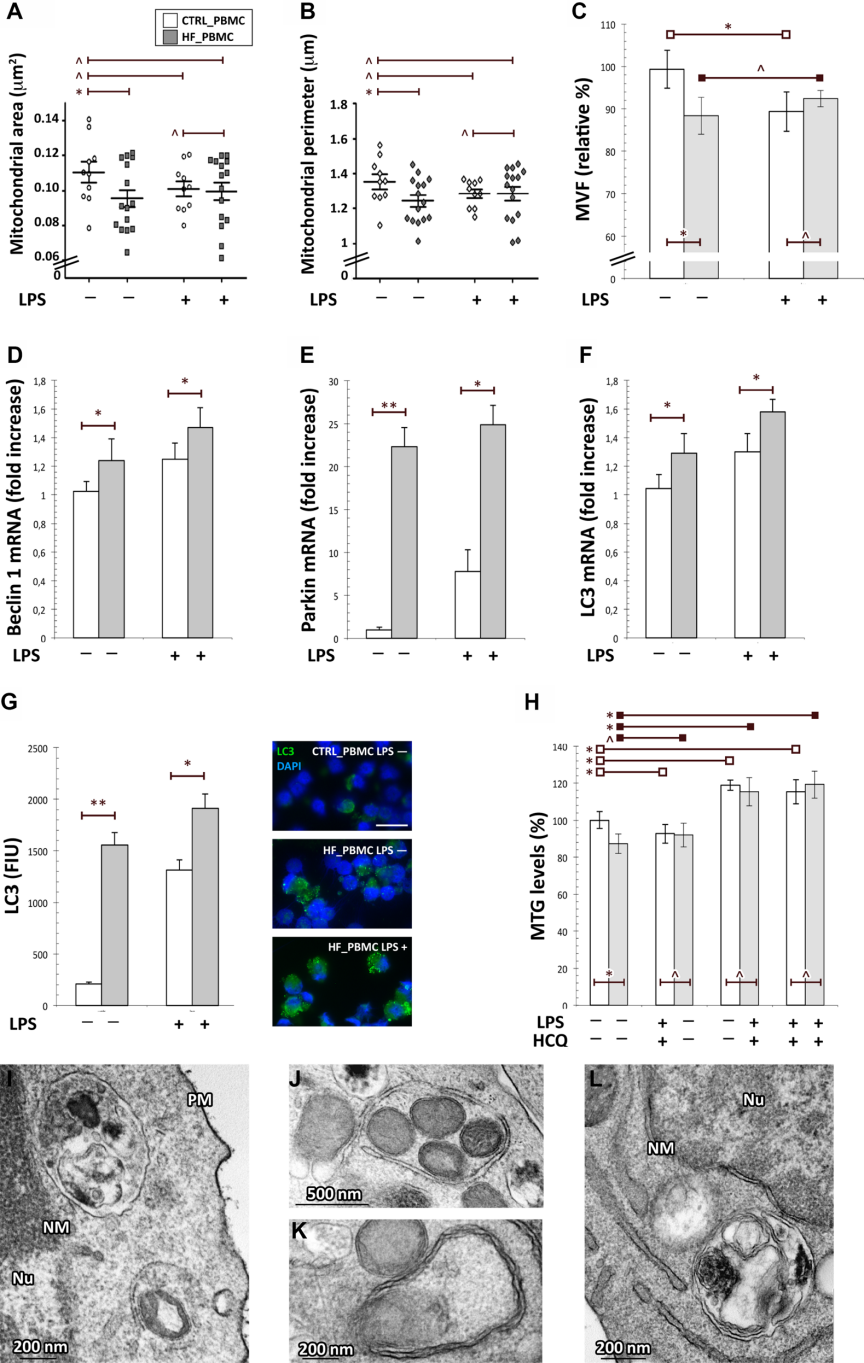
Assessment of mitophagy activation in HF_ and CTRL_PBMCs (**A**–**C**) Morphometric evaluation of mitochondrial structural parameters. At baseline, the HF-PBMCs displayed mitochondria characterized by a significant reduction of both area and perimeter and a total mitochondrial volume density smaller than in CTRL_PBMCs (Figure 4A–4C; ^*^p < 0.05). (**D**–**F**) Analysis of mRNA expression of genes involved in the mitophagic response. The autophagic genes were more expressed in HF_PBMCs with respect to CTRL_PBMCs, and they were further upregulated in response to inflammatory stimulation (Mann–Whitney test: ^*^p < 0.05; ^**^p < 0.01). (**G**) Immunofluorescence assay of LC3 protein expression. bar = 20 μm The quantitative analysis showed a significant increase of the LC3 fluorescence signals in HF_PBMCs, that was even higher in the presence of the proinflammatory stimulus (*T* Student test: ^*^p < 0.05 and ^**^p < 0.01). (**H**) Assessment of mitochondrial mass and mitophagic flux in HF and CTRL_PBMCs. At baseline, the MTG assay showed levels of mitochondrial mass lower in HF_PBMCs compared to CTRL_PBMCs. After LPS-stimulation, while CTRL_PBMCs showed a net reduction of the mitochondrial mass, the HF_PBMCs maintained low levels of mass similar to those of unstimulated cells. The reduction of MTG staining in HF-PBMCs and in the stimulated PBMCs was blocked by treatment with the lysosomal inhibitor HCQ. These data indicate that HF_PBMCs had higher levels of mitophagic flux at baseline, as compared to CTRL_PBMCs, and that the pro-inflammatory stimulus applied to CTRL_PBMCs induced levels of mitophagic flux similar to that of HF_PBMCs at baseline (Student *T* test: ^*^p < 0.05; *p* = NS). (**I**–**L**) Representative micrographs of mitochondria from HF_PBMCs. The ultrastructural analysis showed an extensive autophagy of mitochondria both in the absence of stimulus (I) and after LPS stimulation (J–L). (TEM micrographs, uranyl acetate/lead citrate; Abbreviations: NM, nuclear membrane, PM, plasma membrane, Nu, nucleus).

Indeed, the HF-PBMCs displayed organelles characterized by a significant reduction of the mitochondrial area and perimeter, with a total mitochondrial volume density smaller than in CTRL_PBMCs (Figure 4A–4C; *p <* 0.05).

Then, to verify that oxidative stress and mitochondrial impairment also induce activation of selective autophagy of mitochondria in HF_PBMCs, we analyzed the expression of genes involved in the mitophagic response, such as Beclin 1, Parkin and LC3 [33]. The RT-PCR quantitation showed that the autophagic genes were upregulated in HF_PBMCs, as compared to CTRL_PBMCs, and that they were further upregulated in response to inflammatory stimulation (Figure 4D–4F; *p <* 0.05 to 0.01). In addition, in agreement with the gene expression data, the quantitative immunofluorescence analysis showed a significant increase of the LC3 signals in HF_PBMCs, that was even higher in the presence of the proinflammatory stimulus (*p <* 0.05 to 0.01; Figure 4G).

Next, to verify whether autophagy was specific for mitochondria, we examined mitochondrial mass in HF_ and CTRL_PBMCs. At baseline, the MitoTracker Green (MTG) assay showed levels of mitochondrial mass lower in HF_PBMCs compared to CTRL_PBMCs (Figure 4H; *p <* 0.05). After LPS-stimulation, CTRL_PBMCs showed a net reduction of the mitochondrial mass whereas the HF_PBMCs maintained low levels of mass similar to that of unstimulated cells (Figure 4H: *p* = NS).

Furthermore, the study of the mitophagic flux in the presence of lysosomal inhibitors revealed that the reduction of MTG staining in HF_PBMCs and in all stimulated PBMCs was blocked by the lysosomal inhibitor hydroxychloroquine (Figure 4H, *p <* 0.05). These data indicate that HF_PBMCs had higher levels of mitophagic flux at baseline, as compared to CTRL_PBMCs, and that the LPS-stimulation of the CTRL_PBMCs induced levels of mitophagic flux similar to that of the HF_PBMCs (Figure 4H, *p* = NS).

To confirm the presence of mitochondrial degradation in HF_PBMCs, we performed an ultrastructural analysis that showed an extensive autophagy of mitochondria both in the absence of stimulus and after LPS stimulation (Figure 4I–4L).

Taken together, our results indicate that the mitochondrial dysfunction and the subsequent ultrastructural impairment of mitochondrial morphology in HF_PBMCs was associated with an extensive activation of the mitophagic process, similarly to that observed in the CTRL_PBMCs only under an inflammatory stress status.

## DISCUSSION

The investigation of the pathogenetic mechanisms underlying the development and progression of HF is a fundamental step in order to improve diagnostic and therapeutic approaches. Our study focused on the characterization of one of the most complex mechanism involved in the pathogenesis of HF, the mitochondrial oxidative stress in tissue and blood [2, 34].

In this framework, ROS are derived from endogenous sources such as mitochondrial electron transport chain, nicotinamide adenine dinucleotide phosphate oxidase, nitric oxide synthase and xantine oxidase [35]. Their production, mostly due to circulating leukocytes involved in chronic inflammatory responses, is not adequately counteracted by the antioxidant scavenger systems [36].

In the present study, we focused our attention on a selected population of patients with chronic HF in the attempt to exclude any other conditions that could affect the production of oxidative stress. In PBMCs isolated from these highly selected patients with chronic HF, we evaluated ROS levels, mitochondrial function and morphology, the burden of cell damage and the mitophagic flux. The same evaluations were performed in a group of healthy subjects. Previous studies have shown that high levels of ROS and increased production of superoxide anion by neutrophils are present in the blood of HF patients [29, 37–39] and that white blood cells and platelets producing ROS can amplify oxidative stress and organ damage in HF [24]. Moreover, high concentrations of ROS in PBMCs of HF patients are able to determine the depolarization of mitochondrial structure and to trigger a programmed cell death [40].

As expected, our data show that HF patients have a PBMCs population producing an excess of ROS in relation with reduced mitochondrial functional performance and high scores of ultrastructural damage. The disruption of the inner mitochondrial membrane in these circulating cells, also documented by low IMM/OMM index values, is likely responsible of the electron transport chain impairment and of mitochondrial uncoupling ultimately leading to overproduction of superoxide anion and of other partially reduced species of molecular oxygen.

The activation of intracellular counteracting mechanisms, such as upregulation of antioxidant genes and enhancement of mitophagic flux, appear inadequate to maintain a proper mitochondrial dynamics in these conditions. Consequently, the cell microenvironment is more prone to oxidative stress and becomes trigger of inflammatory responses. Interestingly, the mitochondrial phenotype of the HF_PBMCs is similar to that of the CTRL_PBMCs undergoing experimental conditions that mimic inflammatory stress. As suggested by other studies, it is therefore likely that dysfunctional circulating leukocytes can act as an amplifier of oxidative stress and of cell damage at tissue level, therefore worsening the evolution of HF [22, 24].

A limitation of our study is represented by the limited number of HF patients enrolled for the analysis, as a consequence of the highly selected study criteria. On the other hand, the latter allowed us to avoid any other conditions producing oxidative stress.

In conclusion, our findings are consistent with the notion that HF is associated with an impairment of leukocyte function related to mitochondrial damage, increased oxidative stress and apoptosis [22]. The breakdown of the functional status of circulating leukocytes can contribute to the worsening of HF by promoting the impairment of the host defenses and by favouring the progression of the disease [26].

Finally, we would like to stress the observation that the cytofluorimetric assessment of mitochondrial membrane potential and of mitochondrial ROS in HF_PBMCs, as performed in our study, may represent useful tools for monitoring the oxidative stress at the cellular level, as they reflect the stress within the cardiovascular system [22]. This evidence may also open the way to new diagnostic and therapeutic approaches in HF management. In particular, targeting the mitochondrial dysfunction of circulating PBMCs may be a promising strategy to counteract HF development and progression.

## MATERIALS AND METHODS

### Ethical standards

The study design was approved by the Institutional Review Board of the Sant’Andrea Hospital and Sapienza University of Rome (1916_15) The investigation conforms with the principles outlined in the Declaration of Helsinki. All patients gave their informed written consent to participate to the study.

### Study subjects

We examined 118 consecutive chronic HF patients, referring to the outpatient clinic of the Cardiology unit of Sant’Andrea Hospital in Rome from March 2015 to August 2016. Out of them, we enrolled 15 patients matching the following inclusion criteria: age under 70 years and ejection fraction of the left ventricle below 40% (EF < 40%). The latter was measured with 2D echo Simpson's biplane during mono-dimensional and bi-dimensional echocardiography with a Siemens Acuson C200 apparatus.

The patients with malignancy, inflammatory or infectious diseases, diabetes mellitus or ischemic heart disease, history of cigarette smoking and alcohol abuse were excluded. As a control group (CTRL) we enrolled ten healthy subjects referring to the transfusion center of the same hospital.

### PBMCs preparation and cultures

Venous blood specimens were obtained from both HF patients and CTRL group. Samples were drawn into collection tubes containing EDTA, delivered directly to the Ultrastructural Pathology Lab and immediately processed to isolate PBMCs using density gradient centrifugation over Ficoll-Paque™ PLUS (Amersham Biosciences/GE Healthcare).

PBMCs were cultured in duplicate at the density of 1 × 10^6^ cells/ml on T25 flasks (Becton Dickinson, Oxnard, CA, USA) in RPMI-1640 supplemented with 10% fetal bovine serum and antibiotics. To unmask a possible status of mitochondrial dysfunction, they were treated or not with a standardized proinflammatory stimulus (1 μg/ml Lipopolysaccharide S (LPS; Sigma Chemicals Co.; St. Louis, MD, USA) at 37° C from 3 to 48 hrs) [41], or with a pro-oxidant stimulus (500 μmol Hydrogen Peroxide (H_2_O_2_) for 30 min). Cultured PBMCs were then divided into several aliquots and used for flow cytometry and for the ultrastructural evaluation.

### Assessment of cell damage

Apoptosis was assessed by annexin V-FITC and PI (Annexin V-FITC kit, Miltenyi Biotec GmbH, Bergisch Gladbach, Germany). PBMCs were stained according to the manufacturer's instructions and analyzed with MACSQuant Analyzer (Miltenyi Biotec GmbH). The excitation and emission wavelengths were 488/525 nm (B1 channel) for Annexin V-FITC and 488/655-730 nm (B3 channel) for PI detection. The flow cytometric data were supported by a transmission electron microscopy analysis with quantitative assessment of ultrastructural findings of cellular damage such as apoptotic nuclei, caryorrexis, caryolisis and fragmentation of cellular membranes. For this evaluation, at least 30 different microscopic fields were randomly acquired from ultrathin sections of each PBMCs sample and digitalized at 14,000× original magnification.

### Evaluation of oxidative stress

For intracellular ROS detection, the cells were incubated with 5 μM 2′,7′-dichlorofluorescein diacetate (DCFH-DA, Sigma-Aldrich, St. Louis, MO, USA) for 10 min at 37° C for cytoplasmic oxidative stress assessment, or with 1μM MitoSOX™ Red mitochondrial superoxide indicator (Molecular Probes, Invitrogen, Eugene, OR, USA) for 15 min at 37° C for mitochondrial oxidative stress assessment, protected from light, extensively washed with PBS, re-suspended in pre-warmed medium and collected with MACSQuantH Analyzer flow cytometer (Miltenyi Biotec GmbH). The excitation and emission wavelengths were 488/525 nm (B1 channel) for DCFH-DA and 488/585 nm (B2 channel) for MitoSOX™ Red detection.

### Assessment of antioxidant enzymes

For the superoxide dismutase (SOD) and glutathione peroxidase (GPx) levels, quantitative assays were performed on PBMCs culture supernatants following the manufacturer's instructions by using commercial kits (RANSOD-superoxidodismutase and RANSEL-glutathione peroxidase, Randox, Crumlin, UK).

### Assessment of mitochondrial membrane potential

For mitochondrial membrane potential (Δψm) assessment, the cells were incubated with tetramethylrhodamine methyl ester (TMRM, Molecular Probes, Invitrogen) 500 nM for 30 min at 37° C and with 5, 5′, 6, 6′-tetrachloro-1, 1′, 3, 3′-tetraethylbenzimidazolylcarbocyanine iodide 1.5 μM (JC-1, Molecular Probes) for 30 min at 37° C. The samples were protected from light, washed with warmed PBS, resuspended in pre-warmed medium and collected with MACSQuantH Analyzer flow cytometer. The excitation and emission wavelengths were 488/585 nm (B2 channel) for TMRM analysis and 488/525 to 585 nm (B1 + B2 channels) for JC-1 polychromatic fluorescence emission evaluation.

### Evaluation of mitochondrial mass

Mitochondrial mass was measured by a cytofluorimetric assay upon staining with MitoTracker Green FM (MTG-FM; Molecular Probes, Invitrogen) 50 nM for 30 minutes at 37° C. Excitation wavelength was 488 nm and fluorescence emission was measured at 585 nm (B1 channel).

### Analysis of cytofluorimetric signals

All fluorescence signals obtained from the flow cytometer were analysed by MACSQuantify software (Miltenyi Biotec GmbH) and visualized on a three-decade log scale. The mean fluorescence intensity (MFI ± SE) of TMRM, DCFH-DA and Mito-SOX was calculated from three independent experiments with evaluation of at least 20,000 events for assay.

### Transmission electron microscopy and mitochondrial morphometry assessment

PBMCs treated as above were washed three-times in PBS and fixed with 2% glutaraldehyde in PBS for 2 hours at 4° C. Samples were post-fixed with 1% osmium tetroxide in veronal acetate buffer pH 7.4 for 1 hour at 25° C, stained with uranyl acetate (5 mg/ml) for 1 hour at 25° C, dehydrated in acetone and embedded in Epon 812 (EMbed 812, Electron Microscopy Science, Hatfield, PA, USA). Ultrathin sections obtained with an Ultracut EMFCS ultramicrotome (Leica Microsystems, Wetzlar, Germany) were unstained or poststained with uranyl acetate and lead hydroxide, and examined under a Morgagni 268D Transmission Electron Microscope (TEM) (FEI, Hillsboro, OR, USA) equipped with a Mega View II charge-coupled device camera (SIS, Soft Imaging System GmbH, Munster, Germany).

For the two-dimensional morphometric analysis of mitochondria, at least 20 cell sections in ten different microscopic fields were randomly captured from ultrathin sections of each PBMCs sample and digitalized at 28,000 × original magnification. Area (A) and Perimeter (P) of all mitochondria were measured with the AnalySIS software (Soft Imaging System). The three-dimensional measures of Sphericity and Volume were subsequently derived according to the methods proposed by Noto *et al.* [42].

The sphericity of each structure was expressed by calculating the Shape Factor: $\phi =\frac{\pi \sqrt{\frac{4A}{\pi }}}{p}$.

The Volume (μm^3^) was corrected for Shape Factor and calculated by applying the following formula: $V=\phi \frac{4\pi }{3}{\left(\frac{p}{2\pi }\right)}^{3}$.

The derived mitochondrial volumetric fraction (mitochondrial volume density) was calculated by applying the formula: $MVF=\frac{tmV}{cV}$(tmV: total mitochondrial Volume; cV: cellular Volume).

### Ultrastructural quantitation of mitochondrial damage

At least 30 cell sections, randomly taken in ten different microscopic fields, obtained from ultrathin sections of each PBMCs sample were acquired at 28,000× original magnification and digitalized with a Mega View II charge-coupled device camera (Soft Imaging System).

The micrographs were analyzed with the AnalySIS software (Soft Imaging System) for the percentage of damaged mitochondria classified using a grading scale based on the mitochondrial area with intact cristae, as previously described [41]. Thus, injury grading was categorized into three levels of morphological mitochondria damage: Mt-G3, severe; Mt-G2, moderate and Mt-G1, slight, corresponding to 0%, 1–50% and 51–75% of the area occupied by intact cristae. An additional parameter of the mitochondrial injury was the convolution degeneration related to the inner mitochondrial membrane (IMM) length [13, 14, 43]. All mitochondria observed in 20 ultrathin sections, randomly taken at 56,000 X for all PBMCs samples, were measured for both lengths of IMM and OMM; hence, the IMM/OMM index was calculated for each mitochondrion. Mitochondrial damage was correlated to a low mean value of IMM/OMM index, corresponding to partial or total loss of IMM cristae.

### Immunofluorescence

For the immunofluorescence analysis, PBMCs were fixed with 4% paraformaldehyde followed by treatment with 0.1 M glycine for 20 min at 25° C and with 0.1% Triton X-100 for additional 5 min at 25° C for cell membrane permeabilization. Cells were then incubated with mouse monoclonal anti-LC3 (1:100 in PBS; 5F10 312 Nanotools, Teningen, Germany) for 1 hour at 25° C. The primary antibody was visualized, after appropriate washing with PBS, by using goat anti-mouse IgG–FITC (1:50 in PBS; Cappel Research Products, Santa Ana, CA, USA) for 30 min at 25° C. Nuclei were stained with 4,6-diamidino-2-phenylindole dihydrochloride (DAPI, 1 : 10,000; Sigma Chemicals, St. Louis, MO, USA). Quantitative analysis of the LC3 fluorescence intensity for cytoplasmic area was performed by the analysis of at least 100 cells for each sample in five different fields randomly taken from three independent experiments and performed by the Axiovision software (Zeiss).

### Assessment of mitophagic flux

For the inhibition of the autophagic flux, PBMCs were treated with 30 μM hydroxychloroquine (HCQ, Sigma) for 3 hrs to block lysosomal degradation. Subsequently, they were stimulated or not with LPS 1 μg/ml for 3 hrs. After treatment, PBMCs were divided into several aliquots and they were used for flow cytometry and PCR assays, as described above, and for the mitochondrial morphometry assessment by TEM. The mitophagy flux compares the mitochondrial mass with and without lysosomal inhibitors (PBMCs_HCQ+/PBMCs_HCQ- fluorescence levels) normalized to the corresponding value in control cells. The mitochondrial mass was evaluated by a cytofluorimetric assay (MTG-FM levels) [44, 45] and with a novel morphometric approach (MVF values).

### RNA extraction and cDNA synthesis

RNA was extracted by using the Quick-RNA™ MiniPrep (Zymo Research, Irvine, CA, USA) according to the manufacturer's instructions. Each sample was treated, quantified and 1 μg was used to reverse transcription by using the iScript cDNA synthesis kit (Bio-Rad) with thermal cycling programme as follows: 25° C for 5 min, followed by 46° C for 20 min and 95° C for 1 min.

### Primers

The amplification of the cDNA fragments of target genes expression and of the ribosomal 18S RNA (housekeeping gene) was obtained by using specific oligonucleotides chosen through the online tool Primer-BLAST42 and purchased from Invitrogen (Invitrogen, Carlsbad, CA, USA). Primers list and characteristics are reported in Table 2. For each primer pair, we performed a no-template control which produced negligible signals.

**Table 2:** Primers used for target and housekeeping genes

Gene	Primer sequence
18s	5′-AACCAACCCGGTCAGCCCCT- 3′ (sense), 5′-TTCGAATGGGTCGTCGCCGC- 3′ (antisense)
SOD-2	5′-GGTGGAGAACCCAAAGGGGAGTTG- 3′ (sense) 5′-TTATTGAAACCAAGCCAACCCCAACCT- 3′ (antisense)
Beclin-1	5′-GGATGGTGTCTCTCGCAGAT- 3′ (sense) 5′-TTTCTTGCCCTTCCTTTCTG- 3′ (antisense)
Parkin	5′-GGCTGTGGGTTTGCCTTCT-3′ (sense) 5′-TGCTTCCCAACGAGCCTG-3′ (antisense)
LC3	5′-CGCACCTTCGAACAAAGAG- 3′ (sense) 5′-TTGGCACTTTCTGTGGACAT- 3′ (antisense)

### RT-PCR quantitation

RT-PCR was carried out in 96-well plates by using the iQ SYBR Green Supermix (Bio-Rad) and the iCycler Real-Time Detection System (iQ5; Bio-Rad). The thermal cycling programme was performed as follows: an initial denaturation step at 95° C for 3 min, followed by 40 cycles at 95° C for 10 s and 60° C for 30 s. Melting curves were performed for each primer pair. The relative quantification of gene expression was assessed by comparative threshold cycle method (2^−ΔΔCT^), including normalization of the gene expression to ribosomal 18 S RNA, showing a stable expression in all experimental conditions. All experiments were performed in triplicate and values are reported as mean ± SD.

### Statistical methods

Student's *T* test was used to evaluate statistical significance among variables with a normal distribution. The values are expressed as mean ± SE. Mann–Whitney non-parametric tests was used to compare variables without a normal distribution. The values are expressed as median ± Interquartile Ranges (IR) from three independent experiments. *P* values < 0.05 were assumed as statistically significant.

## References

[1] Mozaffarian D, Benjamin EJ, Go AS, Arnett DK, Blaha MJ, Cushman M, Das SR, de Ferranti S, Després JP, Fullerton HJ, Howard VJ, Huffman MD, Isasi CR, Writing Group Members, and American Heart Association Statistics Committee, and Stroke Statistics Subcommittee (2016). Heart Disease and Stroke Statistics-2016 Update: A Report From the American Heart Association. Circulation.

[2] Grieve DJ, Shah AM (2003). Oxidative stress in heart failure. More than just damage. Eur Heart J.

[3] Rosca MG, Hoppel CL (2013). Mitochondrial dysfunction in heart failure. Heart Fail Rev.

[4] Friedman JR, Nunnari J (2014). Mitochondrial form and function. Nature.

[5] Youle RJ, van der Bliek AM (2012). Mitochondrial fission, fusion, and stress. Science.

[6] Benard G, Bellance N, James D, Parrone P, Fernandez H, Letellier T, Rossignol R (2007). Mitochondrial bioenergetics and structural network organization. J Cell Sci.

[7] Vásquez-Trincado C, García-Carvajal I, Pennanen C, Parra V, Hill JA, Rothermel BA, Lavandero S (2016). Mitochondrial dynamics, mitophagy and cardiovascular disease. J Physiol.

[8] Kanki T, Wang K, Klionsky DJ (2010). A genomic screen for yeast mutants defective in mitophagy. Autophagy.

[9] Youle RJ, Narendra DP (2011). Mechanisms of mitophagy. Nat Rev Mol Cell Biol.

[10] Ikeda Y, Sciarretta S, Nagarajan N, Rubattu S, Volpe M, Frati G, Sadoshima J (2014). New insights into the role of mitochondrial dynamics and autophagy during oxidative stress and aging in the heart. Oxid Med Cell Longev.

[11] Indo HP, Davidson M, Yen HC, Suenaga S, Tomita K, Nishii T, Higuchi M, Koga Y, Ozawa T, Majima HJ (2007). Evidence of ROS generation by mitochondria in cells with impaired electron transport chain and mitochondrial DNA damage. Mitochondrion.

[12] Kubli DA, Gustafsson ÅB (2012). Mitochondria and mitophagy: the yin and yang of cell death control. Circ Res.

[13] Putignani L, Raffa S, Pescosolido R, Aimati L, Signore F, Torrisi MR, Grammatico P (2008). Alteration of expression levels of the oxidative phosphorylation system (OXPHOS) in breast cancer cell mitochondria. Breast Cancer Res Treat.

[14] Putignani L, Raffa S, Pescosolido R, Rizza T, Del Chierico F, Leone L, Aimati L, Signore F, Carrozzo R, Callea F, Torrisi MR, Grammatico P (2012). Preliminary evidences on mitochondrial injury and impaired oxidative metabolism in breast cancer. Mitochondrion.

[15] Levine B, Kroemer G (2009). Autophagy in aging, disease and death: the true identity of a cell death impostor. Cell Death Differ.

[16] Wang K, Klionsky DJ (2011). Mitochondria removal by autophagy. Autophagy.

[17] Hayashi D, Ohshima S, Isobe S, Cheng XW, Unno K, Funahashi H, Shinoda N, Okumura T, Hirashiki A, Kato K, Murohara T (2013). Increased (99m)Tc-sestamibi washout reflects impaired myocardial contractile and relaxation reserve during dobutamine stress due to mitochondrial dysfunction in dilated cardiomyopathy patients. J Am Coll Cardiol.

[18] Ahuja P, Wanagat J, Wang Z, Wang Y, Liem DA, Ping P, Antoshechkin IA, Margulies KB, Maclellan WR (2013). Divergent mitochondrial biogenesis responses in human cardiomyopathy. Circulation.

[19] Kaludercic N, Takimoto E, Nagayama T, Feng N, Lai EW, Bedja D, Chen K, Gabrielson KL, Blakely RD, Shih JC, Pacak K, Kass DA, Di Lisa F, Paolocci N (2010). Monoamine oxidase A-mediated enhanced catabolism of norepinephrine contributes to adverse remodeling and pump failure in hearts with pressure overload. Circ Res.

[20] Redout EM, Wagner MJ, Zuidwijk MJ, Boer C, Musters RJ, van Hardeveld C, Paulus WJ, Simonides WS (2007). Right-ventricular failure is associated with increased mitochondrial complex II activity and production of reactive oxygen species. Cardiovasc Res.

[21] Marín-García J, Akhmedov AT (2016). Mitochondrial dynamics and cell death in heart failure. Heart Fail Rev.

[22] Kong CW, Hsu TG, Lu FJ, Chan WL, Tsai K (2001). Leukocyte mitochondria depolarization and apoptosis in advanced heart failure: clinical correlations and effect of therapy. J Am Coll Cardiol.

[23] Li P, Wang B, Sun F, Li Y, Li Q, Lang H, Zhao Z, Gao P, Zhao Y, Shang Q, Liu D, Zhu Z (2015). Mitochondrial respiratory dysfunctions of blood mononuclear cells link with cardiac disturbance in patients with early-stage heart failure. Sci Rep.

[24] Ijsselmuiden AJ, Musters RJ, de Ruiter G, van Heerebeek L, Alderse-Baas F, van Schilfgaarde M, Leyte A, Tangelder GJ, Laarman GJ, Paulus WJ (2008). Circulating white blood cells and platelets amplify oxidative stress in heart failure. Nat Clin Pract Cardiovasc Med.

[25] Leu HB, Lin CP, Lin WT, Wu TC, Lin SJ, Chen JW (2006). Circulating mononuclear superoxide production and inflammatory markers for long-term prognosis in patients with cardiac syndrome X. Free Radic Biol Med.

[26] Song B, Li T, Chen S, Yang D, Luo L, Wang T, Han X, Bai L, Ma A (2016). Correlations between MTP and ROS Levels of Peripheral Blood Lymphocytes and Readmission in Patients with Chronic Heart Failure. Heart Lung Circ.

[27] Kong CW, Huang CH, Hsu TG, Tsai KK, Hsu CF, Huang MC, Chen LC (2004). Leukocyte mitochondrial alterations after cardiac surgery involving cardiopulmonary bypass: clinical correlations. Shock.

[28] Sam F, Kerstetter DL, Pimental DR, Mulukutla S, Tabaee A, Bristow MR, Colucci WS, Sawyer DB (2005). Increased reactive oxygen species production and functional alterations in antioxidant enzymes in human failing myocardium. J Card Fail.

[29] Hall AR, Burke N, Dongworth RK, Hausenloy DJ (2014). Mitochondrial fusion and fission proteins: novel therapeutic targets for combating cardiovascular disease. Br J Pharmacol.

[30] Mishra P, Chan DC (2016). Metabolic regulation of mitochondrial dynamics. J Cell Biol.

[31] Rovira-Llopis S, Bañuls C, Diaz-Morales N, Hernandez-Mijares A, Rocha M, Victor VM (2017). Mitochondrial dynamics in type 2 diabetes: pathophysiological implications. Redox Biol.

[32] Yoon Y, Krueger EW, Oswald BJ, McNiven MA (2003). The mitochondrial protein hFis1 regulates mitochondrial fission in mammalian cells through an interaction with the dynamin-like protein DLP1. Mol Cell Biol.

[33] Cordero MD, De Miguel M, Moreno Fernández AM, Carmona López IM, Garrido Maraver J, Cotán D, Gómez Izquierdo L, Bonal P, Campa F, Bullon P, Navas P, Sánchez Alcázar JA (2010). Mitochondrial dysfunction and mitophagy activation in blood mononuclear cells of fibromyalgia patients: implications in the pathogenesis of the disease. Arthritis Res Ther.

[34] Givertz MM, Colucci WS (1998). New targets for heart-failure therapy: endothelin, inflammatory cytokines, and oxidative stress. Lancet.

[35] Burgoyne JR, Mongue-Din H, Eaton P, Shah AM (2012). Redox signaling in cardiac physiology and pathology. Circ Res.

[36] Linke A, Adams V, Schulze PC, Erbs S, Gielen S, Fiehn E, Möbius-Winkler S, Schubert A, Schuler G, Hambrecht R (2005). Antioxidative effects of exercise training in patients with chronic heart failure: increase in radical scavenger enzyme activity in skeletal muscle. Circulation.

[37] Díaz-Vélez CR, García-Castiñeiras S, Mendoza-Ramos E, Hernández-López E (1996). Increased malondialdehyde in peripheral blood of patients with congestive heart failure. Am Heart J.

[38] Ellis GR, Anderson RA, Lang D, Blackman DJ, Morris RH, Morris-Thurgood J, McDowell IF, Jackson SK, Lewis MJ, Frenneaux MP (2000). Neutrophil superoxide anion—generating capacity, endothelial function and oxidative stress in chronic heart failure: effects of short- and long-term vitamin C therapy. J Am Coll Cardiol.

[39] White M, Ducharme A, Ibrahim R, Whittom L, Lavoie J, Guertin MC, Racine N, He Y, Yao G, Rouleau JL, Schiffrin EL, Touyz RM (2006). Increased systemic inflammation and oxidative stress in patients with worsening congestive heart failure: improvement after short-term inotropic support. Clin Sci (Lond).

[40] Harris SI, Balaban RS, Barrett L, Mandel LJ (1981). Mitochondrial respiratory capacity and Na+- and K+-dependent adenosine triphosphatase-mediated ion transport in the intact renal cell. J Biol Chem.

[41] Raffa S, Scrofani C, Valente S, Micaloni A, Forte M, Bianchi F, Coluccia R, Geurts AM, Sciarretta S, Volpe M, Torrisi MR, Rubattu S (2017). In vitro characterization of mitochondrial function and structure in rat and human cells with a deficiency of the NADH: ubiquinone oxidoreductase Ndufc2 subunit. Hum Mol Genet.

[42] Noto A, Raffa S, De Vitis C, Roscilli G, Malpicci D, Coluccia P, Di Napoli A, Ricci A, Giovagnoli MR, Aurisicchio L, Torrisi MR, Ciliberto G, Mancini R (2013). Stearoyl-CoA desaturase-1 is a key factor for lung cancer-initiating cells. Cell Death Dis.

[43] Carta S, Tassi S, Delfino L, Omenetti A, Raffa S, Torrisi MR, Martini A, Gattorno M, Rubartelli A (2012). Deficient production of IL-1 receptor antagonist and IL-6 coupled to oxidative stress in cryopyrin-associated periodic syndrome monocytes. Ann Rheum Dis.

[44] Mauro-Lizcano M, Esteban-Martínez L, Seco E, Serrano-Puebla A, Garcia-Ledo L, Figueiredo-Pereira C, Vieira HL, Boya P (2015). New method to assess mitophagy flux by flow cytometry. Autophagy.

[45] Swiader A, Nahapetyan H, Faccini J, D’Angelo R, Mucher E, Elbaz M, Boya P, Vindis C (2016). Mitophagy acts as a safeguard mechanism against human vascular smooth muscle cell apoptosis induced by atherogenic lipids. Oncotarget.

